# One size does not fit all: local determinants of measles vaccination in four districts of Pakistan

**DOI:** 10.1186/1472-698X-9-S1-S4

**Published:** 2009-10-14

**Authors:** Anne Cockcroft, Neil Andersson, Khalid Omer, Noor M Ansari, Amir Khan, Ubaid Ullah Chaudhry, Umaira Ansari

**Affiliations:** 1 CIET in Pakistan, House 226, Block 18, Gulshan-e-Iqbal, Karachi, Pakistan; 2 Centro de Investigación de Enfermedades Tropicales (CIET), Universidad Autónoma de Guerrero, Acapulco, Mexico; 3 Department of geography and urban regional planning, University of Peshawar, Peshawar, Pakistan

## Abstract

**Background:**

Rates of childhood vaccination in Pakistan remain low.There is continuing debate about the role of consumer and service factors in determining levels of vaccination in developing countries.

**Methods:**

In a stratified random cluster sample of census enumeration areas across four districts in Pakistan, household interviews about vaccination of children and potentially related factors with 10,423 mothers of 14,542 children preceded discussion of findings in separate male and female focus groups. Logistic regression analyses helped to clarify local determinants of measles vaccination.

**Results:**

Across the four districts, from 17% to 61% of mothers had formal education and 50% to 86% of children aged 12-23 months had received measles vaccination. Children were more likely to receive measles vaccination if the household was less vulnerable, if their mother had any formal education, if she knew at least one vaccine preventable disease, and if she had not heard of any bad effects of vaccination. Discussing vaccinations in the family was strongly associated with vaccination. In rural areas, living within 5 km of a vaccination facility or in a community visited by a vaccination team were associated with vaccination, as was the mother receiving information about vaccinations from a visiting lady health worker. Focus groups confirmed personal and service delivery obstacles to vaccination, in particular cost and poor access to vaccination services. Despite common factors, the pattern of variables related to measles vaccination differed between and within districts.

**Conclusions:**

Vaccination coverage varies from district to district in Pakistan and between urban and rural areas in any district. Common factors are associated with vaccination, but their relative importance varies between locations. Good local information about vaccination rates and associated variables is important to allow effective and equitable planning of services.

## Background

Coverage of childhood vaccination remains low in many developing countries [1], including Pakistan [2]. In order to increase vaccination rates, it is appropriate first to understand the factors related to vaccination coverage and uptake.

The poorest people in developing countries have lower access to and use of health services, including vaccination, than their better off neighbours [3]. There is continuing debate about the relative importance of parental knowledge and attitudes and service delivery factors (including interaction with the population supposed to be served) as determinants of vaccination rates [4]. Some authors stress parental (demand side) factors, including knowledge, attitudes, education and socio-economic status, as important in determining vaccine coverage, either in demanding vaccinations or in accepting the offer of vaccinations [5-9]. Others emphasize the role of delivery of services (supply side); this includes both the knowledge and attitudes of service providers and their interaction with parents, and availability of vaccination services [10-13]. A study in Colombia found the knowledge of vaccinators influenced vaccination rates in their coverage areas [14].

One argument is to increase coverage of vaccination through a technical programme without tailoring to local circumstances [15]. In developed countries with a relatively good vaccination service offer, parental attitudes tend to determine vaccination uptake. But the importance of parental factors is less clear in developing countries, with relatively poor services or restricted access. The determinants of vaccination may also vary with local cultural and other factors, as well as national and sub-national differences in level and quality of services. Understanding the interplay of factors determining vaccination rates in specific locations is important for local planning of programmes to increase vaccination rates.

As part of a process of building capacities for collecting and using local data for planning public services under devolved local government in Pakistan, together with district personnel we undertook representative household surveys in four districts. These included questions about vaccinations of children under five years old, parental knowledge and attitudes, and household socio-economic status. We linked the household information with information about the sample communities and availability of vaccination services. This allowed us to examine the personal and service-related factors associated with vaccination rates in the four districts, with quite markedly different rates of vaccination.

## Methods

A registered ethical review board in Karachi, Pakistan, approved the social audit programme in Pakistan, including the work in the focus districts, in 2004.

### Sampling

We selected the four districts purposively for the capacity-building initiative: Khairpur in Sindh province, Haripur in North West Frontier province (NWFP), and Khanewal and Sialkot in Punjab province. They joined our initial focus district of Lasbela in southern Balochistan province [16]. In each district the elected district nazim (mayor) had expressed interest in the district social audit process; in some cases the nazim had approached CIET to request technical support for a district and sub-district evidence-based planning process. The districts were not intended to be nationally or provincially representative.

**Figure 1:**
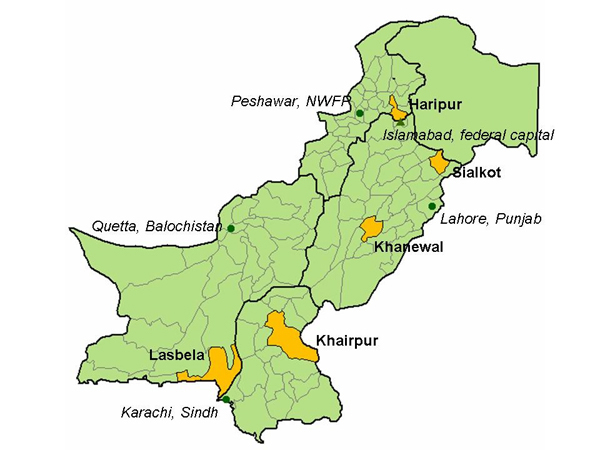
Map of focus districts.

Khairpur is a conservative district in interior Sindh. Large parts of the east and south of the district are desert areas and arid hills, and the district population is mainly concentrated in the west and north. Haripur is close to Islamabad and relatively liberal by NWFP standards. Much of the district is fertile with easy terrain but, like most of the province, it has few urban communities. Khanewal lies towards the less developed south of Punjab; it relies mainly on agriculture and, although quite densely populated, it has relatively few urban sites. Sialkot is located in the more developed north of Punjab and lies close to the borders with India and Kashmir; it is small, densely populated, and heavily industrialized. The map in Figure 1 shows the location of the four districts, together with the initial focus district (Lasbela).

In each district, we drew a stratified random cluster sample. First, we randomly selected union councils (the lowest administrative unit) from each *tehsil* (sub-district administrative area), reflecting the urban/rural spread in each district, and with the number selected according to the population in each *tehsil*. The official list of union councils provided by the district government was the sampling frame for the selection of union councils. From each union council we randomly selected one community (village or *mohalla*) from the list of communities in the union. In each selected community, the sample included a group of 100 contiguous households with children below five years old, spreading out from a random starting point. There was no sampling within the site; all the eligible contiguous households up to 100 were included. 

### Instruments

A *household questionnaire* included: questions to a household respondent about demographics and socioeconomic status of the household; and questions to mothers or caretakers of children below five years old, concerning their education, knowledge, attitudes and practices, as well as vaccination status of the children. We used well-known local terms for the various vaccinations, and described their timing and administration (e.g. "an injection into the arm" for measles vaccine) to assist mothers' recall; we did not attempt to verify the mothers' reports by checking vaccination cards.

The field team leaders completed *community profiles* by means of discussion with a knowledgeable person in each community and their own observations. Each community profile included information about location of health facilities offering vaccinations and whether vaccination teams visited the community.

A *key informant interview* collected information from lady health workers (LHW) in those sites where they worked, including their education and training, visits to the households, any problems they faced, and their relevant knowledge and practice.

We developed *feedback focus group guides*. These presented the findings from the household survey about the actual vaccination rates in the district and, based on this evidence, invited discussion about the perceived reasons for non-vaccination of children, and about how to encourage and support parents to vaccinate children.

### Data collection

Trained field teams from each district, with a majority of female interviewers, undertook data collection during spring and summer 2005. Usually, each team completed the household survey, community profile, and key informant interviews in one community in one day. The field teams took with them a letter from the district government, giving official status to the work. On entering each community, the supervisor explained the purpose of the survey to community leaders and sought their support for the field-work in the community.

After initial analysis of the household findings, which provided evidence about vaccination rates in the district, trained teams returned to the same communities and conducted separate male and female focus group discussions in each community, based on this evidence, using the focus group guides. The participants for the separate male and female groups were drawn from among the house-holds included in the household survey. Each group comprised some 8-12 participants. The trained reporters took notes during the discussions and afterwards, together with the facilitators, prepared reports on the discussions. The focus groups took place in the summer of 2005.

### Data management and analysis

Trained operators undertook data entry using the public domain software package Epi Info. Double data entry with validation reduced keystroke errors. Further cleaning of the dataset looked for logical errors, out of range responses and duplications. The cleaning was completed by checking back to the original data registers as necessary.

Analysis relied on CIETmap software [17]. To investigate the relationship between household socio-economic status and important outcomes, we defined a composite binary variable for household vulnerability, as an indicator of socio-economic status. The vulnerability variable was based on household roof construction, degree of overcrowding, and occupation of the main breadwinner. If at least two out of the three factors were adverse, we categorised the household as "vulnerable". We categorized the remaining households as "less vulnerable". Although for each district the sample size in each *tehsil* reflected the relative population in that *tehsil*, this was not exact. Therefore, to take into account under- and over-sampling between *tehsils*, we calculated weights and applied these when making district level estimates. All the district figures presented here are weighted, unless stated otherwise.

We calculated vaccination rates for each district separately, among children aged 12-23 months. Further analysis examined the associations between childhood vaccination, specifically measles vaccination (among children aged 10-59 months), and related risk and resilience factors, first in a univariate analysis and then in a multiple logistic regression analysis, stepping down from an initial saturated model. Explanatory variables included were those related to the outcome in univariate analysis or for which there was prior reason to believe were likely to be related to the outcome. Initial sequential stratification revealed that the associations between many of the variables and measles vaccination were different between urban and rural communities and between the four districts. An overall logistic regression model, with stratification by district, showed significant interactions between district and other explanatory variables. We therefore undertook separate logistic regression models for urban and rural communities in each of the four districts: eight models overall. In the logistic regression models, we adjusted the 95% confidence intervals around the Odds Ratios to allow for clustering [18,19]. Each model initially included the same explanatory variables, and variables not significantly related to the outcome in this model were sequentially removed to produce the final most parsimonious model explaining the outcome. The explanatory variables included in all the initial models were: mother has some formal education; household not vulnerable; mother can correctly identify a vaccine preventable illness; mother visited by LHW and told about vaccination; mother has not heard of any bad effects of vaccination; mother has discussed immunisation in the family; mother participates in decision about vaccinating the child; government health facility offering immunisation within 5 km; vaccination team visits the community. We eventually had seven models, because data for urban sites in Haripur were too sparse to allow separate analysis. All urban sites in Khairpur, Khanewal, and Sialkot were within 5 km of a government health facility offering vaccination. We report adjusted Odds Ratios from the final models (taking into account the effects of the other variables in the model), together with the cluster-adjusted 95% confidence intervals around the adjusted Odds Ratios.

## Results

### Population characteristics and immunisation rates

Table 1 shows some characteristics of the population in the household sample in each district. Using a common definition, there were more vulnerable households in Khairpur and Khanewal than in Haripur and Sialkot. The proportion of urban households was lowest in Haripur, reflecting the overall rural nature of NWFP. Within Punjab, Sialkot (an industrialized district in the north of the province) had a higher proportion of urban house-holds than Khanewal, towards the south of the province. The proportion of mothers with any formal education varied considerably, from only 17% in Khairpur to as high as 61% in Sialkot.

**Table 1:** The household sample in the four districts.

	Khairpur	Haripur	Khanewal	Sialkot
Number of households	3249	2017	2400	2275
â€˜Vulnerableâ€™ households^a^	51%(1690/3183)	23%(468/1982)	48%(1112/2360)	25% (582/2248)
Households in urban sites	26% (738/3249)	10% (200/2017)	18% (412/2400)	28% (622/2275)
Number of mothers of children under 60 months	3421	2091	2497	2414
Mothers with some formal education	17% (517/3410)	46% (949/2082)	27% (706/2494)	61% (1448/2403)
Number of children <60 months	4739	2682	3586	3535
Number of children 12-23 months	781	512	652	703

^a^The household vulnerability variable is a composite variable intended as an indicator of socio-economic status.The variable is based on three factors:roof construction, crowding, and occupation of the main breadwinner. If two out of the three factors are adverse, the household is categorized as â€˜vulnerableâ€™.

All urban communities were within 5 km of a government facility offering childhood vaccination, except for one of three urban communities in Haripur. Many rural communities were also within this distance (30/70 in Khairpur, 18/28 in Haripur, 25/31 in Khanewal, and 21/28 in Sialkot). Among urban communities, 8/16 were visited by a vaccination team in Khairpur, 1/3 in Haripur, 5/6 in Khanewal, and 7/10 in Sialkot. Among rural communities, visits of vaccination teams were common in Punjab (30/31 communities in Khanewal and 23/27 communities in Sialkot had been visited); visits were less common in NWFP (9/26 communities in Haripur) and Sindh (26/71 communities in Khairpur).

The proportions of children aged 12-23 months reported by their mothers or caretakers to have received different vaccinations are shown in Table 2. In all four districts, the rate of BCG vaccination (the first vaccination) was higher than the rate of the full course of DPT (three doses) and the rate of measles vaccination (the last in the series, given at nine months). Measles vaccination coverage was notably lower in Khairpur and Khanewal than in Haripur and Sialkot. Rates of polio vaccination were high in all districts.

**Table 2:** Immunisation rates in the four districts among children aged 12-23 months.

	Percent (numbers) who received the vaccination			
	
Vaccination	Khairpur	Haripur	Khanewal	Sialkot
BCG	75 (562/775)	90 (452/506)	82 (526/644)	95 (651/686)
DPT full course (3 doses)	52 (371/757)	85 (428/507)	68 (427/637)	88 (602/686)
Measles	50 (356/737)	80 (396/497)	65 (413/635)	86 (590/685)
Polio drops (last 12 m)	99 (760/767)	99 (502/506)	100 (638/639)	100 (681/682)

The rates of measles vaccination by sex of the child and in urban and rural communities in the four districts are shown in Table 3. Vaccination rates were slightly higher among boys in all districts. In all districts, measles vaccination rates were notably higher in urban communities. The lowest rate was in rural communities in Khairpur, while very high rates prevailed in urban sites of Haripur and Sialkot.

**Table 3:** Measles vaccination in the four districts among children aged 12-23 months, by sex of child and residence.

	Percent (numbers) who received the vaccination			
	
Vaccination	Khairpur	Haripur	Khanewal	Sialkot
Measles â€“ all	50 (356/737)	80 (396/497)	65 (413/635)	86 (590/685)
Boys	53(187/370)	82 (215/265)	66 (228/346)	87 (304/348)
Girls	48 (159/350)	79 (181/232)	64 (185/289)	85 (286/337)
urban communities	68 (137/200)	96 (46/48)	70 (82/111)	95 (157/166)
rural communities	42 (219/537)	79 (350/449)	64 (331/524)	83 (433/519)

### Knowledge and attitudes about vaccinations and role of lady health workers

Table 4 shows the knowledge and attitudes of mothers about vaccinations. Nearly all mothers had heard about vaccinations. Between 76% and 88% of mothers could correctly identify at least one vaccine-preventable illness, in response to an open-ended question. Almost all the mothers believed that it was worthwhile to vaccinate children. Virtually all the mothers in all districts who believed it was worthwhile to vaccinate children gave as their reason (in response to an open-ended question) that it protected the children against illness. Among the few who did not think it worthwhile to vaccinate children, the main reasons (common to all four districts) were that it was "not necessary" or that it "made the child sick" afterwards. Between 83% and 91% of mothers reported they had discussed childhood vaccination within the family. Very few mothers said they had heard of any bad effects of childhood vaccination; rather more in Khairpur than in the other three districts. Among those few mothers who had heard of any bad effects, many mentioned actual side effects of vaccination such as fever and pain and swelling at the site (Khairpur 69%, Haripur 25%, Khanewal 37%, Sialkot 40%), while others mentioned fears and misconceptions about side effects, such as that the child could get polio or die, or that vaccination would make the child sterile.

**Table 4:** Knowledge and attitudes about childhood immunisations among mothers.

	Percent (numbers) of mothers			
	
Knowledge/attitude	Khairpur	Haripur	Khanewal	Sialkot
Have heard about childhood immunisations	83 (2760/3400)	94 (1950/2083)	94 (2323/2490)	98 (2365/2408)
Know correctly at least one vaccine preventable illness	76 (2503/3357)	83 (1717/2079)	76 (1855/2459)	88 (2097/2392)
Believe it is worthwhile to immunise children	91 (3047/3374)	97(2007/2087)	96(2387/2495)	99 (2373/2410)
Have heard of bad effects of immunisation	11 (359/3333)	4 (74/2074)	3 (81/2490)	3 (71/2393)
Have discussed immunisation in the family	83 (2733/3334)	91(1874/2067)	84(2064/2485)	89(2102/2371)
Ever visited by LHW	45(1464/3415)	62(1256/2083)	60 (1447/2496)	65 (1541/2411)
Told by LHW about childhood immunisations:				
among those visited	25 (350/1427)	30 (364/1228)	25 (326/1403)	24 (348/1535)
among all mothers	11 (350/3378)	18 (364/2055)	15 (326/2452)	15 (348/2405)

The slightly different denominators for the different variables reflect the different numbers of mothers who responded to the specific questions.

Less than two thirds of mothers had received a visit by a LHW (Table 3). And among those women who had been visited by a LHW, only about a quarter reported that the LHW had given them any information about childhood vaccination. Thus, only a small proportion (11% to 18%) of all women respondents had been visited by a LHW and told by her about vaccinations.

On the other hand, nearly all the LHWs we interviewed (42/46 in Khairpur; 38/40 in Haripur; 24/29 in Khanewal; and 38/41 in Sialkot) reported they told mothers they visited about the importance and benefits of childhood immunisations. Virtually all the LHWs could mention correctly at least one illness preventable by immunisation; fewer of them said they had heard about side effects of vaccination (28/44 in Khairpur, 18/40 in Haripur, 11/29 in Khanewal, and 11/41 in Sialkot). 

### Factors related to measles vaccination

The final models from the logistic regression analyses are shown in Table 5. The models differed between districts but had many variables in common. In general, fewer variables remained in the final models from the urban sites than in the models from the rural sites.

**Table 5:** Final logistic regression models of variables associated with measles vaccination among children aged 10-59 months.

Explanatory variables	Adjusted Odds Ratio (OR)	Cluster-adjusted 95% confidence interval of adjusted OR
**Urban sites in Khairpur district**		
Mother has some formal education	2.02	1.01-4.05
Household not vulnerable	1.55	1.07-2.24
Mother can correctly identify a vaccine preventable illness	2.47	1.04-5.83
Mother has discussed immunisation in the family	4.17	2.55-6.83
**Rural sites in Khairpur district**		
Mother has some formal education	2.00	1.39-2.87
Household not vulnerable	1.73	1.47-2.04
Male child	1.40	1.18-1.64
Mother can correctly identify a vaccine preventable illness	2.44	2.10-2.84
Mother visited by LHW and told about vaccination	3.11	1.82-5.32
Mother has not heard of any bad effects of vaccination	0.44	0.33-0.57
Mother has discussed immunisation in the family	4.05	1.89-8.66
Government health facility offering vaccination within 5 km	2.02	1.45-2.81
Vaccination team visits the community	1.25	1.16-1.36
**Rural sites in Haripur district**		
Mother has some formal education	1.59	1.11-2.27
Household not vulnerable	1.46	1.16-1.85
Mother can correctly identify a vaccine preventable illness	1.81	1.59-2.06
Mother visited by LHW and told about vaccination	1.60	1.14-2.23
Mother has not heard of any bad effects of vaccination	3.05	1.85-5.01
Mother has discussed immunisation in the family	2.54	1.78-3.61
Mother participates in decision about vaccinating the child	1.57	1.21-2.04
Government health facility offering vaccination within 5 km	1.84	1.37-2.48
Vaccination team visits the community	1.48	1.31-1.67
**Urban sites in Khanewal district**		
Mother has some formal education	4.27	1.41-12.90
Mother has not heard of any bad effects of vaccination	7.75	1.43-41.89
Mother has discussed immunisation in the family	3.36	2.00-5.66
**Rural sites in Khanewal district**		
Mother has some formal education	1.79	1.26-2.53
Household not vulnerable	1.77	1.36-2.30
Mother can correctly identify a vaccine preventable illness	2.18	1.99-2.38
Mother visited by LHW and told about vaccination	1.47	1.01-2.14
Mother has not heard of any bad effects of vaccination	2.19	1.27-3.77
Mother has discussed immunisation in the family	3.65	2.68-4.99
Vaccination team visits the community	5.63	5.49-5.77
**Urban sites in Sialkot district**		
Mother participates in decision about vaccinating the child	2.84	1.20-6.74
**Rural sites in Sialkot district**		
Mother has some formal education	1.77	1.15-2.72
Mother can correctly identify a vaccine preventable illness	1.77	1.01-3.09
Mother visited by LHW and told about vaccination	1.80	1.01-3.22
Mother has not heard of any bad effects of vaccination	3.27	1.08-9.92

Data for urban sites in Haripur were too sparse to allow separate analysis.

All urban sites in Khairpur, Khanewal, and Sialkot were within 5 km of a government health facility offering vaccination.The explanatory variables included in all the initial models were: mother has some formal education; household not vulnerable; mother can correctly identify a vaccine preventable illness; mother visited by LHW and told about vaccination; mother has not heard of any bad effects of vaccination; motherhas discussed immunisation in the family; mother participates in decision about vaccinating the child; government health facility offering immunisation within 5 km; vaccination team visits the community.

### Access to vaccination services

In rural sites in Khairpur and Haripur, the presence within 5 km of a government health facility offering vaccination approximately doubled the likelihood that the child had received measles vaccine. In all districts except Sialkot, in rural sites a vaccination team visiting the community also increased the likelihood that the child had received measles vaccine; this effect was much stronger in rural Khanewal. Access was much less important in urban sites. Data were too sparse to allow separate analysis of urban sites in Haripur. In the other three districts, all the urban communities were within 5 km of a government health facility offering vaccination. In urban sites, a visiting vaccination team did not affect the likelihood of a child receiving measles vaccine.

### Household vulnerability

In rural sites, except in Sialkot, a child from a less vulnerable household (that is, a household with a better socio-economic status) was more likely to have received measles vaccine. But in urban sites, household vulnerability was only a significant factor in Khairpur district.

### Mother's education and knowledge

In all districts, in both urban and rural sites, a child of a mother with some formal education was more likely to have received measles vaccine, except in urban sites in Sialkot. In Khanewal, the strength of the association between mother's education and measles vaccination was greater in urban sites (adjusted OR 4.27) than in rural sites (adjusted OR 1.79). In Khairpur the effect was similar in urban sites (adjusted OR 2.02) and rural sites (adjusted OR 2.00).

Everywhere, except in urban sites in Khanewal and Sialkot, children of mothers who could correctly identify a vaccine preventable illness were more likely to have received measles vaccine. On the other hand, in Haripur, Khanewal and Sialkot, children whose mothers did not know of any bad effects of vaccination were more likely to have received measles vaccine. The situation was apparently different in Khairpur, where in urban sites knowing of bad effects was not a significant variable in the final model, while in rural sites, children of mothers who had not heard of any bad effects of vaccination were significantly *less* likely to have received measles vaccine.

In addition to factors of maternal education and knowledge, in rural sites children whose mothers reported being visited by an LHW *and* being told by her about vaccinations were more likely to have received measles vaccine.

### Discussing and making decisions about vaccination

Children whose mothers reported discussing vaccination within the family were more likely to have received measles vaccine, and this was a strong effect in many places, especially in Khairpur, where it had the strongest individual effect on the likelihood of measles vaccination of any of the variables. Only in Sialkot, once other variables were taken into account, was discussing vaccination within the family not significantly related to the likelihood of a child receiving measles vaccine.

The mother's participation in the decision about vaccinating the child was significantly related to the likelihood of the child receiving measles vaccine only in Haripur (rural sites) and in urban sites in Sialkot.

### Sex of the child

While measles vaccination rates were slightly higher among boys than girls (see Table 3), when other variables were taken into account, boys were more likely to receive measles vaccine only in rural sites in Khairpur. 

## Parental reasons for not vaccinating children

We asked mothers of children who were not fully vaccinated the main reason for this. The responses of mothers in urban and rural communities are shown in Table 6. Common reasons were to do with either "carelessness" of the mother or family, or problems with access to vaccination (facilities too far away or teams not visiting). In rural sites access problems predominated, while in urban sites more mothers cited parental carelessness or lack of awareness (except in Haripur with very few responses from rural sites).

## Views from the focus groups

Presented with the evidence about vaccination rates in the district, the separate male and female group participants gave their views about why children are not vaccinated and suggestions for what they believed could improve vaccination rates. The discussions provided qualitative information to give context to the quantitative findings from the household survey.

**Table 6:** Reasons given by mothers of children not vaccinated or not fully vaccinated (among those who gave a reason)^a^.

	Weighted percent (number)							
	
	Khairpur		Haripur		Khanewal		Sialkot	
				
Main reason why child not vaccinated / not fully vaccinated	Urban	Rural	Urban	Rural	Urban	Rural	Urban	Rural
"Carelessness" on part of mother or other family members	19 (56)	7 (143)	15 (2)	27 (126)	17 (27)	9 (95)	51 (29)	36 (125)
Lack of time / no one to take child for vaccinations	8 (23)	5 (98)	8 (1)	10 (45)	9 (11)	7 (74)	12 (7)	12 (37)
Vaccination would harm the child	16 (42)	3 (54)	15 (2)	11 (50)	12 (17)	8 (85)	14 (8)	13 (43)
Lack of awareness about vaccinations or vaccines schedule	8 (21)	4 (81)	31 (4)	11 (55)	11 (14)	11 (110)	4 (2)	6 (20)
Access problems / no nearby facility / no visit of team	33 (95)	68 (1412)		18 (95)	35 (59)	56 (622)	9 (5)	22 (73)
Don't believe in vaccination / it's useless / not our tradition	11 (33)	5 (99)	31 (4)	12 (61)	15 (19)	7 (78)	8 (5)	5 (16)
Family members do not allow child to be vaccinated	2 (7)	1 (23)		9 (38)	1 (2)	1 (8)	2 (1)	4 (12)
Cannot afford vaccination / too poor to vaccinate	2 (6)	8 (167)		2 (11)		2 (16)		2 (8)
Total	100 (283)	100 (2077)	100 (13)	100 (481)	100 (149)	100 (1088)	100 (57)	100 (334)

^a^This was an open-ended question. We coded the responses given by mothers and subsequently further grouped together similar responses.

### Personal factors

Some group participants expressed the view that some parents do not understand the risks of not vaccinating children.

"People are not educated. That's why they are not aware about the dangers of lack of vaccinations." *(male group, Khanewal)*

"Girls are married off at a young age. They don't know anything about these things." *(female group, Sialkot)*

They also suggested that some parents do not want to understand, or that they weighed future risks less heavily than present concerns.

"Some mothers don't want to understand the risks [of non-vaccination]. They say their child is fine and healthy, he doesn't need anything else." *(female group, Khairpur)*

"Our problems are so many that we are only able to think about dangers coming now, not those that seem far away." *(male group, Khanewal)*

However, participants in many groups thought parents understood very well why they should have their children vaccinated; they cited other reasons for these parents not having their children vaccinated. Sometimes they blamed parental carelessness, or negative and fatalistic family attitudes about vaccination and illness.

"People fully understand the risks. But they are careless. They don't bother to take their children for vaccination." *(male group, Khairpur)*

"My mother-in-law says, What kind of children have you produced that they ought to be vaccinated? We were never vaccinated, so why are you behaving so delicately?" *(female group, Khanewal)*

"Whether a child is vaccinated or not, he is bound to get measles once in his lifetime." *(male group, Haripur)*

Some people pointed to concerns about side-effects of vaccines, lack of efficacy of vaccine, or other negative beliefs about vaccines.

"People love their children. They avoid getting them vaccinated because the injections are painful for children." *(male group, Sialkot)*

"Some children have died after getting a vaccine injection. The rest of the mothers around here are now scared." *(female group, Khairpur)*

"To hell with vaccinations. Children get measles even when they are vaccinated against it." *(female group, Sialkot)*

"People are afraid that vaccinations will sterilize their children." *(male group, Khanewal)*

### Other factors

Many people cited poverty as the reason why parents do not vaccinate their children.

"People are poor, but they don't want anyone to know about their condition. They cannot afford to take their children for vaccinations, so they don't." *(female group, Khairpur)*

Participant 1:"We are only bothered about earning 100 rupees a day. We don't have time to think about measles vaccinations."

Participant 2: "People have money to go and watch a movie. Why don't they have money when it comes to their child's health?" *(male group, Haripur)*

Many people complained about problems with access to and experience of vaccination services, whether from visiting vaccination teams or health facilities. It was clear that, in general, people relied on vaccination teams visiting their community, rather than expected to take their children to a health facility outside the community. In many parts of Pakistan women have limited mobility outside their homes.

"We are not allowed to go to health facilities. That's why we cannot have our children vaccinated." *(female group, Khairpur)*

"Vaccination teams don't visit our area. How can anyone blame parents and their children?" *(female group, Khanewal)*

"The government promises to provide free vaccinations. But when the teams get here, they charge us 100 rupees for each injection." *(Male group, Haripur)*

"Teams come and write down false names. That's why some children are not vaccinated." *(Male group, Khanewal)*

"When we go to a health centre, they tell us the vaccine is finished." *(female group, Khanewal)*

"Health workers re-use the same syringe, and charge us money for a new one." *(female group, Sialkot)*

### Increasing vaccination rates

Mostly, participants called for increased access to free vaccination services, saying that only then can parents be expected to get their children vaccinated.

"Teams must go door to door to vaccinate children. Parents who refuse to have their children vaccinated must be fined." *(female group, Sialkot)*

"Two days before the teams are expected to arrive, it should be announced through the mosque so that mothers can be prepared." *(female group, Khanewal)*

"We will take our children for vaccinations if there is better transport available." *(female group, Khairpur)*

## Discussion

In this paper we have used measles vaccination as an indicator of vaccination coverage. Measles vaccination is a single dose in Pakistan, and is the last of the scheduled childhood vaccinations, targeting children nine months old. Typically, coverage with measles vaccination is considerably lower than that with BCG (given at birth), and similar to that of three doses of DPT. Children who do not complete the course of DPT often do not have measles vaccination either.

We used maternal report as our indicator of the vaccination status of children. Some authors from developed countries have suggested that maternal recall is not a good enough indicator of vaccination status compared with health facility records [20,21]. However, a study from Italy found that parental recall alone was similar to other measures of vaccination status and concluded that "verbal recall should be accepted as reasonably reliable in the absence of cards" [22], while in Australia parental recall of measles vaccination coincided as well as vaccination cards with the presence of antibodies [23]. A study in Turkey, taking polio antibodies as the 'gold standard', found that parental recall was more sensitive but less specific than official records [24]. A study in India found that maternal recall under estimated children's vaccination status, but using vaccination cards was not helpful because less than half the mothers had cards and the cards were often incomplete or grossly inaccurate [25]. Our own experience in Pakistan is that vaccination cards are frequently missing or highly inaccurate. Valadez et al. in Costa Rica concluded that maternal recall could be used for estimating vaccination status, especially for younger children and for single dose vaccines [26]. Langsten and Hill in rural Egypt found that mothers reports were later confirmed by card data for at least 83% of children aged 12-23 months [27]. Gareaballah and Loevinsohn found that mothers' reports in the Sudan were accurate and concluded that for both DPT and measles vaccination, reliance on mothers' reports alone gave accurate estimates of vaccination coverage [28]. Goldman and Pebley in Guatemala highlighted the serious problems with service-based data (including vaccination cards) and recommended using mothers' reports to improve estimates of vaccination coverage [29]. Importantly, authors have reported that even if maternal recall may under- or over-estimate vaccination status, this was not related to factors such as maternal education level or poverty status [25,30]. We therefore believe that our reliance on maternal recall of vaccination status is reasonable and is not likely to have introduced bias into the analysis of factors related to vaccination in the four districts.

The four districts included in this study had measles vaccination coverage rates ranging from 50% up to 86%, in the context of a reported national measles vaccination rate of 60% in 2006-07 [2]. National surveys have reported considerable variation across Pakistan in coverage with measles vaccination and other vaccinations [2,31]. For example, the 2006-07 Demographic and Health Survey reported measles vaccination rates of 51% in Sindh, 54% in Balochistan, 57% in NWFP, and 65% in Punjab [2]. Even these provincial figures mask considerable variation. Our focus districts have relatively good rates for their provinces; Khairpur has about the average rate for Sindh, Haripur is well above the NWFP average, Khanewal is at the Punjab average, and Sialkot is above the Punjab average. Even average district figures do not tell the whole story; in Khairpur there was as much as 26% difference in measles vaccination coverage between urban and rural communities, while the national urban-rural difference is reported to be about 10% [2]. A survey of immunisation coverage in a district of India also found big differences between *panchayats*, with higher coverage in urban and peri-urban *panchayats *[32].

Despite the important differences in overall measles vaccination rates between the four districts, we found common factors associated with measles vaccination. Mother's education was related to measles vaccination in all four districts, in urban and rural sites. Many authors have noted mother's education as an important determinant of childhood vaccination in developing countries [6,9,33,34]. The big difference in the proportion of mothers with any formal education between Khairpur and Sialkot may go some way towards explaining their very different vaccination rates.

The poor are less likely to access health care [3] and less likely to be covered by vaccination programmes [35]. Overall increases in measles vaccination in developing countries in the 1990s have often been accompanied by an increase in the gap between the rich and poor [36]. In a study in Bangladesh, higher socio-economic status was associated with childhood vaccination [33]. We found that children from less vulnerable households (our indicator of better socio-economic status) were more likely to be vaccinated. In Sialkot, the richest district among the four, and in urban sites in Khanewal, we did not find that household vulnerability was associated with vaccination, once other variables were taken into account. This might indicate that when the overall level of services is better and access to services is easier, socio-economic status becomes less of a barrier to vaccination. Discussions in our focus groups confirmed the importance of poverty as a barrier to vaccination in many cases, as parents described being unable to afford the costs of the supposedly "free" immunisations: travel costs, opportunity costs, and demands for unofficial payments. A study of a measles epidemic in Mexico described the out of pocket costs associated with vaccination and also the way the poorest households bore disproportionately the costs of not vaccinating [37].

Some authors have documented a greater likelihood of vaccination among male children [6,33,35]. However, we found that when other variables were taken into account, the sex of the child was associated with vaccination only in rural areas of Khairpur.

Maternal knowledge about vaccine preventable illnesses was quite strongly associated with vaccination in virtually all locations in our study. But knowledge of benefits is not enough by itself to ensure vaccination. Some focus group participants suggested that lack of knowledge of the benefits of vaccination or the risks of non-vaccination were reasons why children were not vaccinated, as has been reported from elsewhere [38], but many others felt failure to vaccinate children happened despite parents being aware of the benefits. Fear of adverse effects of vaccination is cited as a reason for children not being vaccinated, in both qualitative and quantitative studies [9,39] and we found that children of mothers who did not report having heard of bad effects of vaccines were more likely to be vaccinated, in most places. However, this variable had the opposite effect in rural areas of Khairpur; perhaps in this area where very few women have any formal education, having heard of any bad effects equates with being more knowledgeable overall about vaccinations. In urban Khanewal, on the other hand, there was a strong negative effect of having heard about bad effects of vaccines; perhaps here there have been strongly negative stories about vaccination incidents that have discouraged some parents from vaccinating their children.

We have proposed an expansion of the standard knowledge, attitudes, practice (KAP) approach to behaviour change, known as CASCADA [40]. This partial order of causality includes a number of intermediate steps between conscious knowledge and action, the step immediately preceding action being "discussion". In three of the four districts, the mother discussing vaccination in the family was the variable most strongly associated with the child being vaccinated against measles.

While these "demand-side" factors are important in determining vaccination rates, "supply-side" factors are also relevant. Fewer variables remain in the final models from urban sites in the four districts, compared with rural sites. In particular, variables indicating access to services are prominent determinants of vaccination in rural sites: whether there is a government facility providing vaccinations within 5 km of the community and whether a vaccination team visits the community. Qualitative studies have reported that parents cite problems with services as the main obstacles to having their children vaccinated [11,39,41,42]. In this study, groups of both male and female parents mentioned a number of problems with the provision of vaccination services that made it difficult for them to have their children vaccinated.

A positive supply-side factor was visits from LHWs. This positive effect was apparent in rural areas in all four districts. A study of vaccination rates in Bangladesh also found that children living in areas visited by family welfare assistants were more likely to be vaccinated [33] and in Thailand children in families who had contact with village health volunteers were more likely to be vaccinated [10]. A systematic review of lay health workers found a significant benefit of lay health worker interventions to promote vaccination uptake in both children and adults [43].

The pattern of factors affecting vaccination uptake has been reported to differ between countries [44]. Our findings show how it can differ between and even within districts. For district governments planning interventions to increase vaccination services equitably, these local differences matter. For example, in Khanewal dealing with negative perceptions about vaccine side-effects would be important. In Khairpur, extending programmes to reach and inform uneducated women could have an important role. These activities would be in addition to efforts to improve service access, for example through visiting vaccination teams. Further analysis can indicate the effects of combinations of variables [45] and the potential benefits, in terms of gains in numbers of children vaccinated, of different interventions and combinations of interventions [17]. This information about potential gains, together with information about costs of different interventions can assist district planners to make rational decisions about funding priorities.

## Conclusions

There are large variations in childhood vaccination rates between districts and even within districts in Pakistan. While many of the variables associated with vaccination are common across different localities, their relative importance varies. Access to services is a more important factor in rural areas. Parents report difficulties with services as important reasons for not vaccinating children. The pattern of variables related to vaccination varies between and within districts; effective and equitable planning of vaccination services will differ between districts, based on evidence of this sort. It would be of interest to see if similar analyses of factors related to vaccination coverage in other countries reveal the same degree of local heterogeneity.

## List of abbreviations used

CIET: Community Information for Empowerment and Transparency; LHW: Lady health worker; NWFP: North West Frontier Province.

## Competing interests

The authors declare that they have no competing interests.

## Authors' contributions

AC designed the survey, undertook the analysis and drafted the report; NA reviewed the design, supported the analysis and reviewed the report; KO was responsible for the survey in Khairpur and for data management, supported all the district surveys, and reviewed the report; NMA helped manage the surveys in all districts and reviewed the report; AK was responsible for the survey in Haripur and reviewed the report; UUC was responsible for the surveys in Khanewal and Sialkot and reviewed the report; and UA assisted with the surveys, especially the focus groups, was responsible for extracting from the focus group reports, and reviewed the report.
